# Supplementary material for: A transcriptomic continuum of differentiation arrest identifies myeloid interface acute leukemias with poor prognosis
Source: Leukemia. 2020 Jul 13;35(3):724–36. doi: 10.1038/s41375-020-0965-z (PMC7932917; doi:10.1038/s41375-020-0965-z)
Supplement: Supplementary file 1 — Supplemental Data [file 41375_2020_965_MOESM1_ESM.pdf]

SUPPLEMENTAL DATA

A transcriptomic continuum of differentiation arrest identifies myeloid interface acute leukemias with poor prognosis

Bond *et al*

Contents:

- **Supplementary Table Legends**
- **Supplementary Tables S6, S8 - S11**
- **Supplementary Figures S1 - S4**
- **Supplementary Methods**
- **Supplementary References**

Supplementary Table Legends:

Supplementary Table S1: Gene-sets used for GSEA analysis in this study (see Excel file).

Supplementary Table S2: Details of patient cohort (see Excel file).

Supplementary Table S3: Differential gene expression analysis comparing AML-like T-ALLs with other T-ALLs (see Excel file). Positive values denote genes with higher expression in AML-like T-ALLs.

Supplementary Table S4: Differential gene expression analysis for the comparisons of thymic subset populations indicated in each tab (see Excel files).

Supplementary Table S5: ICGS output (see Excel file). First row contains sample names, second row the ICGS clusters, and the following rows guide genes and their normalized expression. The second column indicates the guide genes groups as indicated by the black and white bars in Figure 2A (first tab), Supplementary Figure S3A (second tab (1)) and S3C (third tab (2)).

Supplementary Table S6: Genes included in the targeted NGS panel.

Supplementary Table S7: Mutational status by NGS (see Excel file). 0 = no mutation, 1 = mutation of known significance, 2 = mutation of unknown significance.

Supplementary Table S8: List of genes used for IAL score

Supplementary Table S9: Impact of IAL score on outcome according to ELN subgroup.

Supplementary Table S10: Comparison of clinicobiological characteristics and mutational profiles of cases with high and low IAL scores in the ALFA-0701 cohort (3).

Supplemental Table S11: Univariate analyses of Overall Survival in the ALFA-0701 cohort (3).

Supplementary Table S6: Genes included in the targeted NGS panel.

Gene	Transcript	CCDS	Description
AKT1	ENST00000554581	CCDS9994	v-akt murine thymoma viral oncogene homolog 1
ASXL1	ENST00000375687	CCDS13201	additional sex combs like 1 (Drosophila)
ATM	ENST00000278616	CCDS31669	ataxia telangiectasia mutated
BCL11B	ENST00000345514	CCDS9949	B-cell CLL/lymphoma 11B (zinc finger protein)
BCOR	ENST00000342274	CCDS14250	BCL6 corepressor
CARD11	ENST00000396946	CCDS5336	caspase recruitment domain family, member 11
CCR4	ENST00000330953	CCDS2656	chemokine (C-C motif) receptor 4
CD58	ENST00000457047	CCDS44199	CD58 molecule
CEBPA	ENST00000498907	CCDS54243	CCAAT/enhancer binding protein (C/EBP), alpha
CNOT3	ENST00000406403	CCDS12880	CCR4-NOT transcription complex, subunit 3
CSNK1A1	ENST00000377843	CCDS47303	casein kinase 1, alpha 1
CTCF	ENST00000264010	CCDS10841	CCCTC-binding factor (zinc finger protein)
CUL3	ENST00000264414	CCDS2462	cullin 3
CXCR4	ENST00000409817	CCDS33295	chemokine (C-X-C motif) receptor 4
DDX3X	ENST00000399959	CCDS43931	DEAD (Asp-Glu-Ala-Asp) box helicase 3, X-linked
DNM2	ENST00000359692	CCDS32907	dynamin 2
DNMT3A	ENST00000264709	CCDS33157	DNA (cytosine-5-)-methyltransferase 3 alpha
EED	ENST00000263360	CCDS8273	embryonic ectoderm development
EP300	ENST00000263253	CCDS14010	E1A binding protein p300
ETV6	ENST00000396373	CCDS8643	ets variant 6
EZH2	ENST00000320356	CCDS5891	enhancer of zeste homolog 2 (Drosophila)
FAS	ENST00000355740	CCDS7393	Fas cell surface death receptor
FBXW7	ENST00000263981	CCDS3778	F-box and WD repeat domain containing 7, E3 ubiquitin protein ligase
FLT3	ENST00000241453	CCDS31953	fms-related tyrosine kinase 3
FYN	ENST00000354650	CCDS5094	FYN oncogene related to SRC, FGR, YES
GATA3	ENST00000379328	CCDS31143	GATA binding protein 3
HACE1	ENST00000262903	CCDS5050	HECT domain and ankyrin repeat containing E3 ubiquitin protein ligase 1
HNRNPA2B1	ENST00000356674	CCDS5397	heterogeneous nuclear ribonucleoprotein A2/B1
HRAS	ENST00000417302	CCDS7699	Harvey rat sarcoma viral oncogene homolog
IDH1	ENST00000415913	CCDS2381	isocitrate dehydrogenase 1 (NADP+), soluble
IDH2	ENST00000330062	CCDS10359	isocitrate dehydrogenase 2 (NADP+), mitochondrial
IKZF1	ENST00000349824	CCDS69299	IKAROS family zinc finger 1 (Ikaros)
IL7R	ENST00000303115	CCDS3911	interleukin 7 receptor
IRF4	ENST00000380956	CCDS4469	interferon regulatory factor 4
JAK1	ENST00000342505	CCDS41346	Janus kinase 1
JAK3	ENST00000458235	CCDS12366	Janus kinase 3
KIT	ENST00000288135	CCDS3496	v-kit Hardy-Zuckerman 4 feline sarcoma viral oncogene homolog
KMT2A	ENST00000534358	CCDS55791	lysine (K)-specific methyltransferase 2A
KMT2D	ENST00000301067	CCDS44873	lysine (K)-specific methyltransferase 2D
KRAS	ENST00000311936	CCDS8702	Kirsten rat sarcoma viral oncogene homolog
LEF1	ENST00000379951	CCDS47122	lymphoid enhancer-binding factor 1
NF1	ENST00000358273	CCDS42292	neurofibromin 1
NOTCH1	ENST00000277541	CCDS43905	notch 1
NRAS	ENST00000369535	CCDS877	neuroblastoma RAS viral (v-ras) oncogene homolog
PHF6	ENST00000332070	CCDS14639	PHD finger protein 6
PIK3CA	ENST00000263967	CCDS43171	phosphatidylinositol-4,5-bisphosphate 3-kinase, catalytic subunit alpha
PIK3R1	ENST00000521381	CCDS3993	phosphoinositide-3-kinase, regulatory subunit 1 (alpha)
POT1	ENST00000357628	CCDS5793	protection of telomeres 1
PTEN	ENST00000371953	CCDS31238	phosphatase and tensin homolog
PTPN11	ENST00000351677	CCDS9163	protein tyrosine phosphatase, non-receptor type 11
PTPN6	ENST00000456013	CCDS44821	protein tyrosine phosphatase, non-receptor type 6
PTPRD	ENST00000381196	CCDS43786	protein tyrosine phosphatase, receptor type, D
RB1	ENST00000267163	CCDS31973	retinoblastoma 1
RELN	ENST00000428762	CCDS47680	reelin
RHOA	ENST00000418115	CCDS2795	ras homolog family member A
RPL10	ENST00000424325	CCDS14746	ribosomal protein L10
RPL5	ENST00000370321	CCDS741	ribosomal protein L5
RUNX1	ENST00000344691	CCDS42922	runt-related transcription factor 1
SETD2	ENST00000409792	CCDS2749	SET domain containing 2
SF3B1	ENST00000335508	CCDS33356	splicing factor 3b, subunit 1, 155kDa
SH2B3	ENST00000341259	CCDS9153	SH2B adaptor protein 3
STAT3	ENST00000264657	CCDS32656	signal transducer and activator of transcription 3 (acute-phase response factor)
STAT5B	ENST00000293328	CCDS11423	signal transducer and activator of transcription 5B
SUZ12	ENST00000322652	CCDS11270	SUZ12 polycomb repressive complex 2 subunit
TAL1	ENST00000294339	CCDS547	T-cell acute lymphocytic leukemia 1
TBL1XR1	ENST00000457928	CCDS46961	transducin (beta)-like 1 X-linked receptor 1
TDRD6	ENST00000544460	CCDS55017	tudor domain containing 6
TET2	ENST00000540549	CCDS47120	tet methylcytosine dioxygenase 2
TET3	ENST00000409262	CCDS46339	tet methylcytosine dioxygenase 3
TP53	ENST00000420246	CCDS45606	tumor protein p53
WT1	ENST00000332351	CCDS7878	Wilms tumor 1
ZEB1	ENST00000446923	CCDS44370	zinc finger E-box binding homeobox 1
ZRSR2	ENST00000307771	CCDS14172	zinc finger (CCCH type), RNA-binding motif and serine/arginine rich 2

Supplementary Table S8: List of genes used for IAL score.

CD34	*MAN1A1*
LDLRAD4	*LY9*
ATP10A	*RBM8A*
TSPAN7	*DYRK3*
STARD9	*GUCY1A3*
SMAD1	*KAT6A*
BAALC	*TAB2*
ZMIZ1	*MBTD1*
LOC105373495	*STT3B*
KMT2A	*CD109*
NPR3	*PRKACB*
MLLT3	*CELF2*
BBX	*UBXN4*
SLC9A7	*PRKCE*
FOXN3	*CEP68*
MN1	*SLC25A30*
PSIP1	*SHANK3*
SDK2	*SETBP1*
AFF3	*AKAP2*
RPL13A	*ITGA4*
UPF2	*SMURF2*
GBP4	*ATP2B4*
ITM2C	*ZNF251*
FLJ10038	*MTHFD1*
IFI16	*SPRY1*
C5orf56	*RPS6*
MEF2A	*ELK4*
AKT3	*PRR5L*
KIAA0754	*C2CD2*
CD200	*TLK1*
CLSTN1	*RNF125*
B4GALT6	*DDHD1*
KIAA0125	*UBASH3B*
CD2AP	*CHRDL1*
TMTC4	*NCOA7*
GNB5	*LOC646778*
KLHL13	*GLS*
TTC3	*SUMO4*
ABHD17B	*SLC38A1*
DCK	*GNG7*
PROM1	*HMGB1*
PIK3C2B	*TSPAN5*
ABCB1	*SH3RF1*
LINC01181	*PTAR1*
XYLT1	*GPATCH11*
SPTBN1	*SEC61A2*
SLC38A2	*PROSER2*
KATNAL1	*NOL4L*
GNAI1	*INPP4B*
RUNX3	*ERG*

Supplementary Table S9: Impact of IAL score on outcome according to ELN subgroup.

ELN-2010 Subgroup	Patients, N	HR in High IAL score	95% CI	P values
Favorable	36	0.87	0.31-2.39	0.78
Intermediate	97	1.89	1.15-3.09	0.011
Adverse	43	1.46	0.70-3.06	0.31

According to the ELN-2010 classification (4) that was in use during the ALFA-0701 trial, both Intermediate-1 and Intermediate-2 subgroups were considered in a single Intermediate subgroup. 16 non-classifiable patients were excluded from the analysis.

Supplementary Table S10: Comparison of clinicobiological characteristics and mutational profiles of cases with high and low IAL scores in the ALFA-0701 cohort (3).

	Low IAL score	High IAL score	P values
**Patients, N**	96	96	-
**Median age, years (range)**	62.0 years (50-70)	62.4 years (50-70)	0.55
**WBC, G/L (range)**	6.9 (0.15-187)	4.9 (0.5-211)	0.68
**CD33 expression <70%, N/tested (%)**	17/67	31/70	0.031
**Cytogenetic risk, N (%)**			0.003
Favorable	1 (1%)	3 (3%)	-
Intermediate	72 (75%)	57 (59.5%)	-
Adverse	12 (12.5%)	31 (32.5%)	0.002
NA	11 (11.5%)	5 (5%)	-
**ELN RISK, N (%)**	-	-	0.005
Favorable	22	14	-
Intermediate	51	46	-
Adverse	12	31	0.002
Not classifiable	11	5	-
**High LSC17 score, N (%)**	36 (37.5%)	60 (62.5%)	<0.001
**GENE MUTATIONS, N mutated/tested (%)**	-	-	-
NPM1	43/96	17/94	<0.001
FLT3-ITD	19/96	14/95	0.45
IDH1	8/85	12/84	0.35
IDH2	13/85	11/84	0.83
DNMT3A	26/85	20/84	0.39
TET2	15/85	12/84	0.68
WT1	3/85	6/84	0.33
ASXL1	8/85	10/84	0.63
RUNX1	6/85	17/84	0.014
sAML-type gene mutations *	20/85	38/84	0.004

*sAML = secondary AML type mutations, including *ASXL1, SRSF2, STAG2, BCOR, U2AF1, EZH2, SF3B1* and/or *ZRSR2* (Lindsley *et al*. Blood 2015) (5)

Supplementary Table S11: Univariate analyses of Overall Survival in the ALFA-0701 cohort (3).

Variable	Patients, N	HR	95% CI	P values
GO arm	278	0.82	0.61-1.10	0.19
Age (continuous variable)	278	1.02	0.99-1.05	0.16
WBC (continuous variable)	277	1.003	0.99-1.01	0.071
Adverse cytogenetics*	249	2.89	2.06-4.06	**<0.001**
High CD33 expression (≥70%)	200	0.86	0.60-1.23	0.41
ELN Risk[$]	249	2.19	1.71-2.80	**<0.001**
High LSC17 score	192	2.45	1.71-3.53	**<0.001**
NPM1 mutation	274	0.67	0.48-0.94	0.019
FLT3-ITD mutation	275	1.06	0.72-1.57	0.76
RUNX1 mutation	232	1.11	0.73-1.70	0.62
sAML-type gene mutations[#]	232	1.17	0.85-1.62	0.34
High IAL score	**192**	**1.73**	**1.21-2.46**	**0.002**

GO = Gemtuzumab Ozogamicin.

*Patients with cytogenetic failure were excluded, leaving 58 adverse and 191 favorable/intermediate cases for analysis.

[$]According to the ELN-2010 classification (4) that was in use during the ALFA-0701 trial, both Intermediate-1 and Intermediate-2 subgroups were considered in a single Intermediate subgroup. 16 non-classifiable patients were excluded from the analysis.

[#]sAML = secondary AML type mutations, including *ASXL1, SRSF2, STAG2, BCOR, U2AF1, EZH2, SF3B1* and/or *ZRSR2* (5).

Covariates with significant differences (highlighted in bold) were selected for multivariate analyses, with additional retention of GO treatment arm (Table 1 in main manuscript).

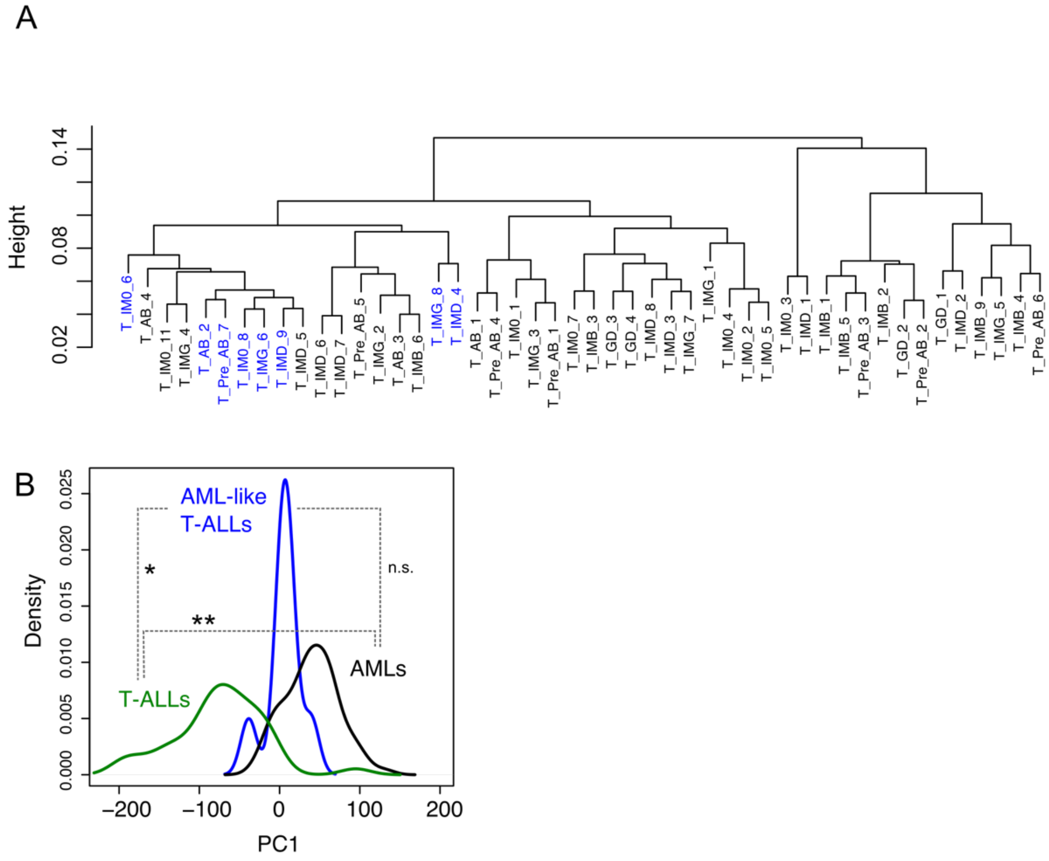

Supplementary Figure S1: Hierarchical clustering and Principal Component Analysis. (A) Unsupervised hierarchical clustering (HC) of the transcriptional profiles of the 48 T-ALL samples in the patient cohort. AML-like cases identified by HC in Figure 1A are indicated in blue. **(B)** Principal Component Analysis (PCA) of the sample cohort of T-ALLs and AMLs. Density of distribution of samples in each group along PC1 is indicated. *: $p<0.05$ and **: $p<0.01$ by Kruskal-Wallis test.

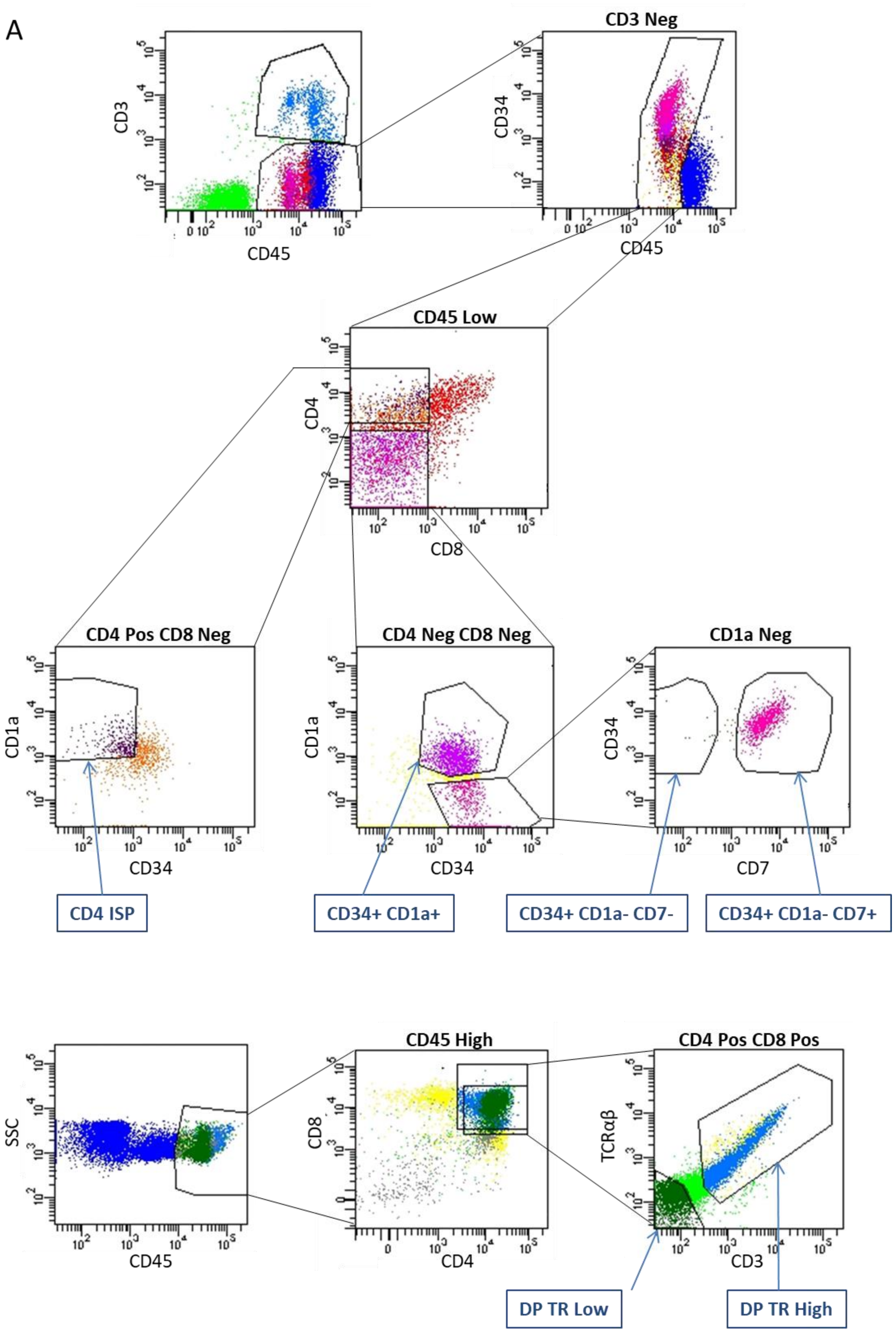
A
CD3 Neg
CD45 Low
CD4 Pos CD8 Neg
CD4 Neg CD8 Neg
CD1a Neg
CD4 ISP
CD34+ CD1a+
CD34+ CD1a- CD7-
CD34+ CD1a- CD7+
CD45 High
CD4 Pos CD8 Pos
DP TR Low
DP TR High
CD3
CD45
CD34
CD4
CD8
CD1a
CD7
SSC
TCRαβ

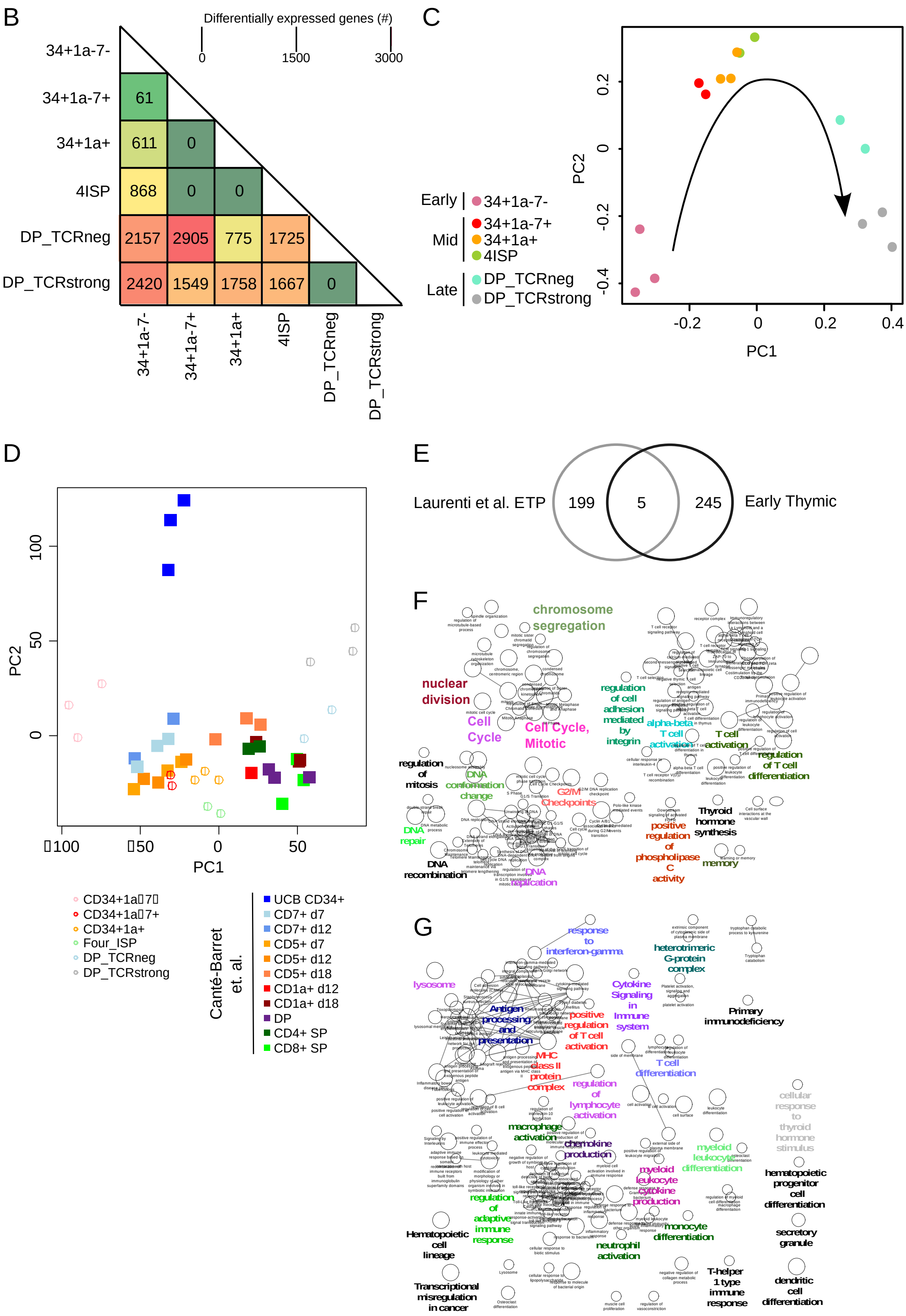
B
Differentially expressed genes (#)
0
1500
3000
34+1a-7-
34+1a-7+
34+1a+
4ISP
DP_TCRneg
DP_TCRstrong
61
611
0
868
0
0
2157
2905
775
1725
2420
1549
1758
1667
0
C
PC2
PC1
-0.2
0
0.2
0.4
-0.4
Early
34+1a-7-
Mid
34+1a-7+
34+1a+
4ISP
Late
DP_TCRneg
DP_TCRstrong
D
PC2
PC1
100
50
0
−100
−50
CD34+1a−7−
CD34+1a−7+
CD34+1a+
Four_ISP
DP_TCRneg
DP_TCRstrong
Canté-Barret et. al.
UCB CD34+
CD7+ d7
CD7+ d12
CD5+ d7
CD5+ d12
CD5+ d18
CD1a+ d12
CD1a+ d18
DP
CD4+ SP
CD8+ SP
E
Laurenti et al. ETP
199
5
245
Early Thymic
F
chromosome segregation
nuclear division
Cell Cycle
Cell Cycle, Mitotic
regulation of cell adhesion mediated by integrin
alpha-beta T cell activation
T cell activation
regulation of T cell differentiation
regulation of mitosis
DNA conformation change
G2/M Checkpoints
DNA repair
DNA recombination
DNA replication
positive regulation of phospholipase C activity
Thyroid hormone synthesis
memory
G
response to interferon-gamma
heterotrimeric G-protein complex
lysosome
Antigen processing and presentation
Cytokine Signaling in Immune system
positive regulation of T cell activation
Primary immunodeficiency
MHC class II protein complex
regulation of lymphocyte activation
T cell differentiation
cellular response to thyroid hormone stimulus
macrophage activation
chemokine production
myeloid leukocyte differentiation
myeloid leukocyte cytokine production
hematopoietic progenitor cell differentiation
regulation of adaptive immune response
monocyte differentiation
Hematopoietic cell lineage
neutrophil activation
secretory granule
Transcriptional misregulation in cancer
T-helper 1 type immune response
dendritic cell differentiation

Supplementary Figure S2: Transcriptional profiling of human thymic subpopulations. (A) Flow cytometry sorting strategy for the indicated subpopulations isolated from human neonatal thymi. **(B-C)** Transcriptional profiling of CD34+ CD1a-CD7- (n=3), CD34+CD1a-CD7+ (n=2), CD34+CD1a+ (n=3), CD4+ISP (n=2), Double Positive (DP) T-receptor (TR) Low (n=2), DP TR High (n=3) populations sorted as in **(A). (B)** number of differentially expressed genes (FDR<0.05 by limma) in pairwise comparisons between indicated populations. **(C)** PCA of indicated populations (based on most variable genes across all thymic populations, see methods). **(D)** Comparison of the gene expression profiles of the thymic populations analyzed here with those of T-cells generated *in vitro* from CB CD34+ cells (6) on a 2D PCA. **(E)** Venn diagram showing overlap of genes in the Laurenti *et al*. ETP geneset (7) and the Early Thymic geneset identified here. Only 5 genes were found in both genesets: *MX1, LGMN, IRF8, CXCR3* and *OAS2*. **(F)** and **(G)** ClueGO pathway analysis of genes unique to **(F)** the Laurenti *et al* ETP signature and **(G)** the Early Thymic geneset identified here. Only genesets with FDR<0.05 are shown.

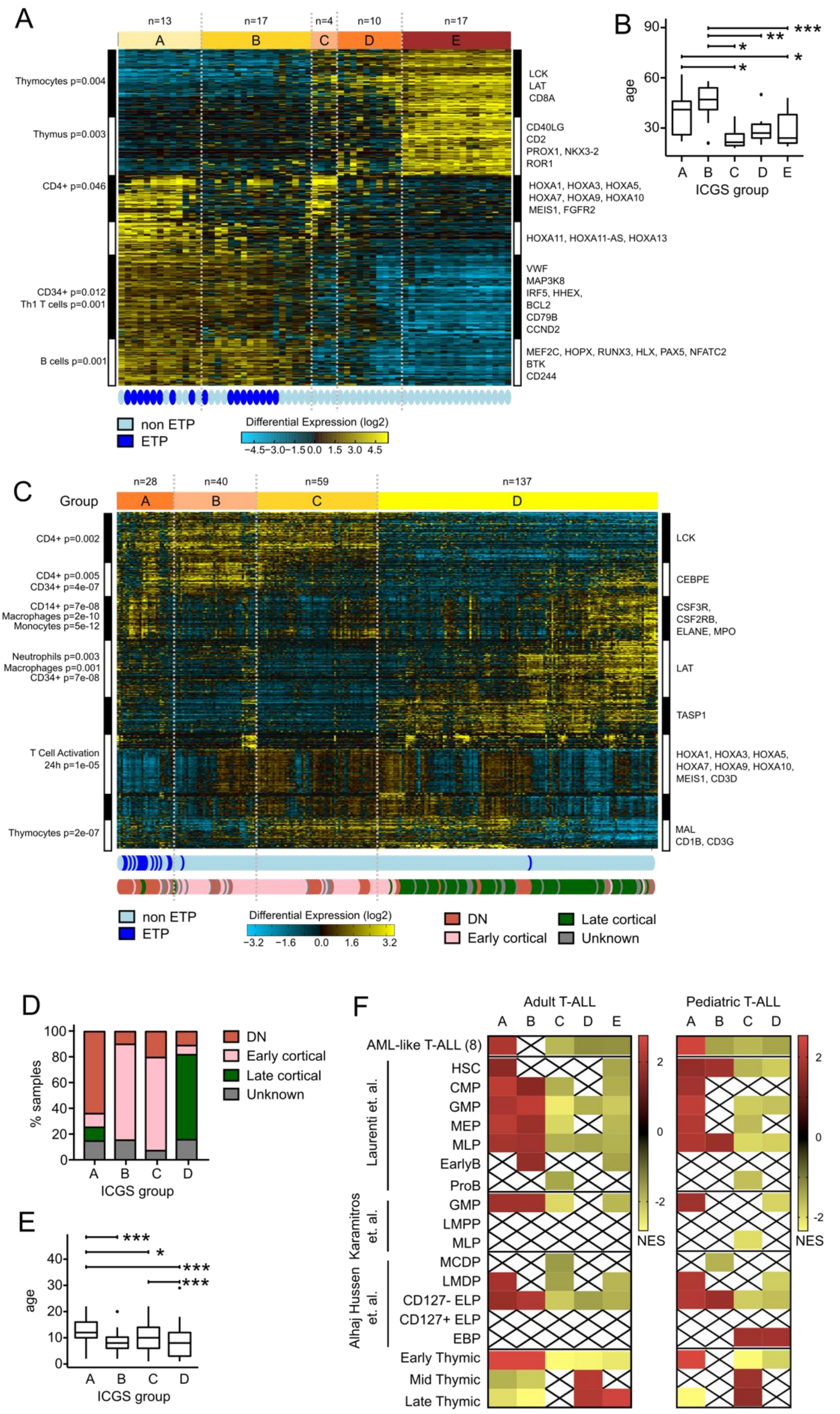
A
n=13
n=17
n=4
n=10
n=17
A
B
C
D
E
Thymocytes p=0.004
Thymus p=0.003
CD4+ p=0.046
CD34+ p=0.012
Th1 T cells p=0.001
B cells p=0.001
LCK
LAT
CD8A
CD40LG
CD2
PROX1, NKX3-2
ROR1
HOXA1, HOXA3, HOXA5,
HOXA7, HOXA9, HOXA10
MEIS1, FGFR2
HOXA11, HOXA11-AS, HOXA13
VWF
MAP3K8
IRF5, HHEX,
BCL2
CD79B
CCND2
MEF2C, HOPX, RUNX3, HLX, PAX5, NFATC2
BTK
CD244
non ETP
ETP
Differential Expression (log2)
−4.5 −3.0 −1.5 0.0 1.5 3.0 4.5
B
age
90
60
30
***
**
*
*
*
A B C D E
ICGS group
C
Group
n=28
n=40
n=59
n=137
A
B
C
D
CD4+ p=0.002
CD4+ p=0.005
CD34+ p=4e-07
CD14+ p=7e-08
Macrophages p=2e-10
Monocytes p=5e-12
Neutrophils p=0.003
Macrophages p=0.001
CD34+ p=7e-08
T Cell Activation
24h p=1e-05
Thymocytes p=2e-07
LCK
CEBPE
CSF3R,
CSF2RB,
ELANE, MPO
LAT
TASP1
HOXA1, HOXA3, HOXA5,
HOXA7, HOXA9, HOXA10,
MEIS1, CD3D
MAL
CD1B, CD3G
non ETP
ETP
Differential Expression (log2)
−3.2 −1.6 0.0 1.6 3.2
DN
Early cortical
Late cortical
Unknown
D
% samples
100
80
60
40
20
0
A B C D
ICGS group
DN
Early cortical
Late cortical
Unknown
E
age
40
30
20
10
0
***
*
***
***
A B C D
ICGS group
F
Adult T-ALL
A B C D E
Pediatric T-ALL
A B C D
AML-like T-ALL (8)
Laurenti et. al.
HSC
CMP
GMP
MEP
MLP
EarlyB
ProB
Karamitros et. al.
GMP
LMPP
MLP
Alhaj Hussen et. al.
MCDP
LMDP
CD127- ELP
CD127+ ELP
EBP
Early Thymic
Mid Thymic
Late Thymic
2
0
-2
NES
2
0
-2
NES

Supplementary Figure S3: ICGS classifies T-ALL samples based on their stage of leukemic differentiation arrest. (A-B) ICGS analysis of adult samples in the Chen *et al*. dataset (1) (n=61 samples, pediatric samples were excluded). **(A)** Heatmap of expression of guide genes selected by ICGS. Genes are represented in rows. White and black bars on the side represent blocks of correlated genes and selected enriched gene ontology groups for these genes are shown. Columns represent individual T-ALL samples. Bottom bars indicate the phenotype of each individual sample, top bar indicates the clusters identified by ICGS. **(B)** Patient age distribution in indicated ICGS groups. * $p<0.05$, ** $p<0.01$ and *** $p<0.001$ by one-way ANOVA with multiple comparisons. Boxes indicate median, interquartile range and whiskers the 95 percentile. **(C-E)** ICGS analysis of pediatric samples in the Liu *et al*. dataset (2) (n=264 samples). **(C)** Heatmap of expression of guide genes selected by ICGS. ETP phenotype and stage of differentiation arrest (as determined by mutation analysis in (2)) are indicated in the bottom bars, top bar indicates the major clusters identified by ICGS. **(D)** Distribution of stages of differentiation arrest in indicated ICGS groups. **(E)** Patient age distribution in indicated ICGS groups. * $p<0.05$ and *** $p<0.001$ by one-way ANOVA with multiple comparisons. Boxes indicate median, interquartile range and whiskers the 95 percentile. **(F)** Enrichment of indicated normal hematopoietic progenitor transcriptional signatures by GSEA. Samples in the indicated ICGS clusters were compared to all other samples in each cohort. NES = Normalized Enrichment Score, crossed out boxes indicate gene-sets that are not significantly enriched (FDR > 0.05).

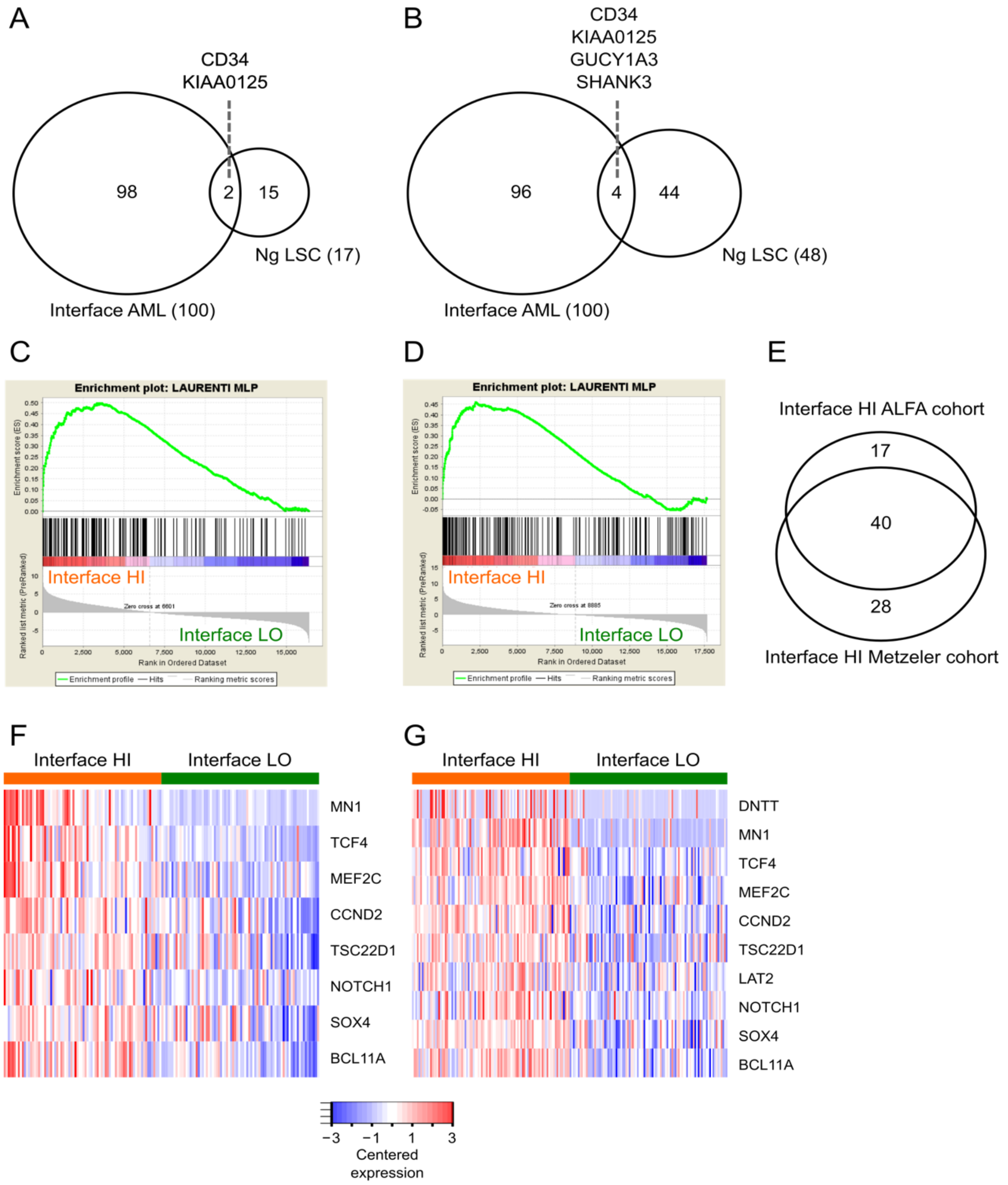

Supplementary Figure S4: AMLs with high IAL scores are distinct from LSC-AML and are enriched for lymphoid transcriptional signatures. (A) Overlap of IAL score with LSC17 score and **(B)** the extended 48 gene list from the same publication (8). Enrichment of MLP signatures (7) in Interface High cases by GSEA in the **(C)** Metzeler *et al* (9) and **(D)** ALFA-1701 (3) studies. These cohorts had overlap in significant differential expression of B-lymphoid genes from the MLP signature, as shown in the Venn diagram in **(E)** and in the heatmaps in **(F)** Metzeler *et al* (9) and **(G)** ALFA-1701 (3).

Supplementary Methods:

Microarray experiments: RNA was extracted from acute leukemia and normal thymic samples using either the RNeasy Micro or Mini Kits (Qiagen), depending on cell numbers. Biotinylated double strand cDNA targets were prepared from 0.3 to 35 ng of total RNA using the NuGEN Ovation Pico WTA System V2 Kit (Cat # 3302) followed by the NuGEN Encore Biotin Module Kit (Cat # 4200) according to manufacturer recommendations. Following fragmentation, 4.5 µg of cDNAs were hybridized for 16 hours at 45^0C, 60 rpm on Human GeneChip® HG-U133 plus 2.0 arrays (Affymetrix). The chips were washed and stained in the GeneChip® Fluidics Station 450 (Affymetrix) using the FS450_0004 script and scanned with the GeneChip® Scanner 3000 7G (Affymetrix) at a resolution of 1.56 µm. Raw data (.CEL Intensity files) were extracted from the scanned images using the Affymetrix GeneChip® Command Console (AGCC) version 4.1.2. CEL files were further processed with Affymetrix Expression Console software version 1.4.1 to calculate probeset signal intensities, using Robust Multi-array Average (RMA) algorithms with default settings.

Next-generation sequencing: Nextera XT (Illumina) DNA Libraries were prepared according to the manufacturer's instructions and sequenced using the Illumina MiSeq sequencing system. The custom NGS panel (Supplementary Table S4) was originally inspired by the repertoire of genes found to be preferentially altered in pediatric ETP-ALL (10), and we have previously reported analyses of other T-ALL cohorts using this panel (11, 12). Sequencing reads were analyzed using institutional software for alignment and mutation calling (Polyweb, Institut Imagine, Paris). Variant calling required ≥50 total reads including ≥10 alternative reads and additional visual confirmation in Integrative Genomics Viewer (https://software.broadinstitute.org/software/igv/). Variants were further filtered by reference to both constitutional (dbSNP, https://www.ncbi.nlm.nih.gov/snp/, ExAC, http://exac.broadinstitute.org/, The 1000 Genomes Browser, https://www.internationalgenome.org/1000-genomes-browsers/) and somatic (COSMIC https://cancer.sanger.ac.uk/cosmic) databases, and by prediction of mutational effect using the Polyphen (http://genetics.bwh.harvard.edu/pph2/), SIFT (https://sift.bii.a-star.edu.sg/) and Cadd (https://cadd.gs.washington.edu/score) tools.

Data comparison with published datasets: Expression data from the LT-HSC, MLP, CMP, GMP, MEP, earlyB, proB umbilical cord blood (CB) populations from Laurenti *et al*(7) (GSE42414) were used to determine a list of highly variable genes across all umbilical cord blood populations (CB-HVGs, 7271 genes, defined as genes differentially expressed between any 2 populations). Microarray data from these genes was extracted from the AML/T-ALL dataset and was combined with the selected samples from GSE42414, batch corrected (using the *ComBat* function from the *sva* package) and normalised (*normalize.quantiles* function from the preprocessCore v1.34.0 package). PCA analysis was performed as above on these CB-HVGs for normal CB populations and T-ALL samples. Expression data from samples in Cante-Barret *et al* (6) (GSE79379) was combined with data from thymic populations, AMLs and T-ALLs profiled here and batch corrected (using the *ComBat* function from the *sva* package). Combined PCA analysis was performed as described in the main methods section.

Isolation of thymic subpopulations: Informed consent was given for provision of normal human thymi removed during neonatal cardiac surgery at Hôpital Necker-Enfants Malades. Mononuclear cell suspensions were obtained by dissection and irrigation of thymic tissue, followed by Ficoll gradient centrifugation. Subpopulations were isolated by fluorescence activated cell sorting (FACS Aria, Becton Dickinson) using the strategy shown in Supplementary Figure S3. The following antibodies were used: CD1a FITC NA1/34 Dako F714101-2; CD3 AF700 UCHT1 BD Biosciences 557943; CD3 APC UCHT1 BD Biosciences 555335; CD34 APC 8G12 BD Biosciences 345804; CD4 V450 (Horizon) RPA-T4 BD Biosciences 560345; CD4 V450 (Horizon) RPA-T4 BD Biosciences 560345; CD45 V500 (Horizon) HI30 BD Biosciences 560777; CD7 PE M -T701 BD Biosciences 332774; CD8 PE-Cy7 SK1 BD Biosciences 335822; TCR alpha / beta PE IP26A Beckman Coulter B49177; TCR gamma / delta PE 11F2 BD Biosciences 333141. For thymic subpopulations CD34+CD1a-CD7-, CD34+CD1a-CD7+, CD34+CD1a+ and CD4+ ISP, FACS sorting was preceded by CD3+ cell depletion using magnetic activated cell sorting (MACS, Miltenyi Biotec).

Calculation of IAL score*:* An AML interface leukemia probeset was defined as the top 100 probes (ranked by t statistic) differentially expressed between AML samples of ICGS cluster 2 and 3 (interface AMLs) and those in ICGS clusters 4 and 5. The interface score for each sample in each of the 2 independent cohorts (3, 9) was calculated as the Sum of

mean-centered log2 values for each of the genes in the AML interface leukemia probeset (71 genes in (9), 82 in (3)). Patient samples in each cohort were split into interface HI and interface LO based on whether their individual interface score was above or below a threshold, set as the median of the interface score for that cohort (median interface score: -5.12 in (9), -10.17 in (3). The robustness of the approach was tested by varying this threshold: similar prognostic results were obtained for any threshold between -15 and 6 for (9), and -80 and 92 in (3).

Outcome analyses: Post-hoc analyses of Overall (OS) and Event-free survival (EFS) of patients with AML were performed on publicly available data (9) and on the ALFA-1701 cohort that we have previously reported (3). Statistical analyses and survival curves were calculated in R with the *survival* package functions *survfit*, *survdiff* and *coxph* (Cox model). For clinicobiological comparisons of IAL High and Low cases in the ALFA-1701 cohort, ELN risk was defined according to the ELN-2010 classification (4), and secondary AML-type genes were defined as previously reported (5). Cytogenetic subgroups were defined as previously described (3), briefly: **Favorable** included t(8;21) and inv(16)/t(16;16); **Adverse** included monosomy 5 or del(5q), monosomy 7 or del(7q), t(6;11), t(9;22), 3q26 abnormalities (except t(3;5)), 11q23 abnormalities (except t(9;11)), and complex karyotypes with 3 abnormalities or more; while the **Intermediate** group included all other anomalies as well as normal karyotypes. Covariates selected for multivariate analyses (Table 1) were selected based on the results of univariate analyses (Supplementary Table S10), with additional retention of GO (gemtuzumab ozogamicin) treatment arm.

Supplementary References:

1. Chen B, Jiang L, Zhong ML, Li JF, Li BS, Peng LJ, et al. Identification of fusion genes and characterization of transcriptome features in T-cell acute lymphoblastic leukemia. Proc Natl Acad Sci U S A. 2018;115(2):373-8.
2. Liu Y, Easton J, Shao Y, Maciaszek J, Wang Z, Wilkinson MR, et al. The genomic landscape of pediatric and young adult T-lineage acute lymphoblastic leukemia. Nat Genet. 2017;49(8):1211-8.
3. Castaigne S, Pautas C, Terre C, Raffoux E, Bordessoule D, Bastie JN, et al. Effect of gemtuzumab ozogamicin on survival of adult patients with de-novo acute myeloid leukaemia (ALFA-0701): a randomised, open-label, phase 3 study. Lancet. 2012;379(9825):1508-16.
4. Dohner H, Estey EH, Amadori S, Appelbaum FR, Buchner T, Burnett AK, et al. Diagnosis and management of acute myeloid leukemia in adults: recommendations from an international expert panel, on behalf of the European LeukemiaNet. Blood. 2010;115(3):453-74.
5. Lindsley RC, Mar BG, Mazzola E, Grauman PV, Shareef S, Allen SL, et al. Acute myeloid leukemia ontogeny is defined by distinct somatic mutations. Blood. 2015;125(9):1367-76.
6. Cante-Barrett K, Mendes RD, Li Y, Vroegindeweij E, Pike-Overzet K, Wabeke T, et al. Loss of CD44(dim) Expression from Early Progenitor Cells Marks T-Cell Lineage Commitment in the Human Thymus. Front Immunol. 2017;8:32.
7. Laurenti E, Doulatov S, Zandi S, Plumb I, Chen J, April C, et al. The transcriptional architecture of early human hematopoiesis identifies multilevel control of lymphoid commitment. Nature immunology. 2013;14(7):756-63.
8. Ng SW, Mitchell A, Kennedy JA, Chen WC, McLeod J, Ibrahimova N, et al. A 17-gene stemness score for rapid determination of risk in acute leukaemia. Nature. 2016;540(7633):433-7.
9. Metzeler KH, Hummel M, Bloomfield CD, Spiekermann K, Braess J, Sauerland MC, et al. An 86-probe-set gene-expression signature predicts survival in cytogenetically normal acute myeloid leukemia. Blood. 2008;112(10):4193-201.
10. Zhang J, Ding L, Holmfeldt L, Wu G, Heatley SL, Payne-Turner D, et al. The genetic basis of early T-cell precursor acute lymphoblastic leukaemia. Nature. 2012;481(7380):157-63.
11. Bond J, Graux C, Lhermitte L, Lara D, Cluzeau T, Leguay T, et al. Early Response-Based Therapy Stratification Improves Survival in Adult Early Thymic Precursor Acute Lymphoblastic Leukemia: A Group for Research on Adult Acute Lymphoblastic Leukemia Study. J Clin Oncol. 2017;35(23):2683-91.
12. Bond J, Touzart A, Lepretre S, Graux C, Bargetzi M, Lhermitte L, et al. DNMT3A mutation is associated with increased age and adverse outcome in adult T-acute lymphoblastic leukemia. Haematologica. 2019.
